# The validity of a professional competence tool for physiotherapy students in simulation-based clinical education: a Rasch analysis

**DOI:** 10.1186/s12909-016-0718-x

**Published:** 2016-08-05

**Authors:** Belinda K. Judd, Justin N. Scanlan, Jennifer A. Alison, Donna Waters, Christopher J. Gordon

**Affiliations:** 1 Faculty of Health Sciences and Sydney Nursing School, University of Sydney, Sydney, Australia; 2 Faculty of Health Sciences, University of Sydney, Sydney, Australia; 3 Sydney Nursing School, University of Sydney, Sydney, Australia

**Keywords:** Competency, Health professional education, Physiotherapy, Rasch model, Simulation

## Abstract

**Background:**

Despite the recent widespread adoption of simulation in clinical education in physiotherapy, there is a lack of validated tools for assessment in this setting. The Assessment of Physiotherapy Practice (APP) is a comprehensive tool used in clinical placement settings in Australia to measure professional competence of physiotherapy students. The aim of the study was to evaluate the validity of the APP for student assessment in simulation settings.

**Methods:**

A total of 1260 APPs were collected, 971 from students in simulation and 289 from students in clinical placements. Rasch analysis was used to examine the construct validity of the APP tool in three different simulation assessment formats: longitudinal assessment over 1 week of simulation; longitudinal assessment over 2 weeks; and a short-form (25 min) assessment of a single simulation scenario. Comparison with APPs from 5 week clinical placements in hospital and clinic-based settings were also conducted.

**Results:**

The APP demonstrated acceptable fit to the expectations of the Rasch model for the 1 and 2 week clinical simulations, exhibiting unidimensional properties that were able to distinguish different levels of student performance. For the short-form simulation, nine of the 20 items recorded greater than 25 % of scores as ‘not-assessed’ by clinical educators which impacted on the suitability of the APP tool in this simulation format.

**Conclusion:**

The APP was a valid assessment tool when used in longitudinal simulation formats. A revised APP may be required for assessment in short-form simulation scenarios.

**Electronic supplementary material:**

The online version of this article (doi:10.1186/s12909-016-0718-x) contains supplementary material, which is available to authorized users.

## Background

Simulation-based education and assessment has been an integral part of medical and nursing curricula for well over a decade [1, 2], with more recent adoption in physiotherapy and other allied health programs [3–6]. The opportunities created by simulation for deliberate, repeated practice of clinical skills in a safe environment have influenced the growing popularity of simulation in health professional education [7]. The support for simulation in physiotherapy and other allied health programs has been strengthened in Australia by government initiatives to increase simulation across the country to help ease the burden of sourcing clinical placements [8]. Simulation in physiotherapy education commonly involves simulating clinical practice for teaching and learning purposes. Clinical situations are created largely using standardized patients but can also use mannequins, part-task trainers, or computer-generated simulations [1]. Standardized patients are healthy people trained to portray individuals with a particular medical condition [7]. Two recent randomized controlled trials concluded that replacing a proportion (up to 25 %) of clinical placement time with simulation did not affect physiotherapy student learning outcomes [9, 10]. In light of these results, by 2014, 16 of the 19 physiotherapy programs across Australia embedded some form of simulation into curricula [8].

There is a growing body of evidence outlining the development, testing and implementation of formal assessment tools for learners in simulation in the fields of medicine and nursing. For example, the Anesthetists Non-Technical Skills (ANTS) tool has been widely adopted for use in simulation to assess non-technical skills such as teamwork among anesthetists [11]. Similarly, the non-technical skills of surgical teams were assessed in simulation by the revised Non-technical Skills Evaluation Tool (NOTECHS) tool which covers domains such as communication and situational awareness [12]. The Mayo High Performance Teamwork Scale was developed for the assessment of teamwork skills of resident doctors and nurses in simulation and has been widely adopted to assess teamwork performance. Competency has been assessed using the Queen’s Simulation Assessment tool with emergency medicine postgraduate trainees in simulation. This tool assesses global clinical competency during a simulation-based Objective Structured Clinical Exam (OSCE) [13]. All of the above assessment tools have demonstrable reliability and validity in different simulation settings [11–13]. A recent systematic review of simulation-based assessments in health [14] found that the use of assessments in simulation settings are effective, although the review highlighted that further research was needed particularly if simulation was used as the only form of assessment. The review studies were predominantly from medicine, further highlighting the need for more research in physiotherapy and other health professions.

There is a lack of assessment tools measuring clinical competence in physiotherapy simulation-based education [15, 16]. Costello and colleagues [17] examined the content validity of a tool to measure physiotherapy student performance with standardized patients using an expert consensus approach with thirty physiotherapy academics and educators. The tool, based on professional standards published by the American Physical Therapy Association was deemed a valid form of measurement as it aligned closely with a consensus of practice expectations. Similarly, Panzarella and colleagues [4] examined the reliability and validity of an assessment tool to measure physiotherapy student performance with standardized patients. This preliminary study was unable to confirm a high level of overall reliability and validity using a four-point rating scale, although there was some support for the content validity of the tool. The focus of the study appeared to be on oral communication skills and clinical reasoning, and not on overall professional competence inclusive of technical skills. There are, however studies involving physiotherapy students that have validated the OSCE format for measuring student competence where a combination of written/video and standardized patient assessment stations were used [18, 19]. The OSCE assessment format and environment is often in a controlled, classroom setting and thus does not compare to the assessment of professional competence during authentic clinical placements in hospitals or other health settings or simulation-based clinical education, making comparisons difficult.

The Assessment of Physiotherapy Practice (APP) is an assessment tool measuring pre-registration physiotherapy students’ professional competence on clinical placements [14]. The APP was originally designed for use by educators to assess student performance over a period of time, usually a clinical placement of 4–6 weeks. This type of assessment is termed ‘longitudinal assessment’ in this paper. A longitudinal assessment is determined using a cumulative approach based on repeated observations of student performance over a pre-defined period, for example, 1 week. This assessment format differs from the assessment of a single-isolated performance, such as that which occurs using the OSCE format. The APP is based on the Australian Standards for Physiotherapy Practice [20], and a large multisite trial across nine universities demonstrated that the APP was a reliable and valid measure of physiotherapy student performance in clinical placements [21, 22]. The APP has been adopted as the primary form of assessment for clinical placement performance across university physiotherapy programs in Australia and New Zealand, and recently was trialed in Canada [23]. Despite this, the construct validity and suitability of the APP for use in simulation-based settings has not been evaluated. It is conceivable that the APP may not demonstrate adequate construct validity as originally shown in clinical placements when assessing student professional competency in simulation. Additionally, it is unknown whether the degree of authenticity of simulation may be a contributing factor to whether the APP is valid in a simulation setting [24]. Since the simulation environment differs from that in which the APP was originally validated, it is important to determine the validity of the APP in simulation [17].

To the best of our knowledge, no tools have been validated for use in the assessment of overall professional competency of physiotherapy students engaged in simulation-based clinical education. The widespread adoption of the APP in simulation in Australia provided the opportunity to examine the validity of the APP in this environment. Thus, the aim of the study was to determine if the APP was a valid and suitable assessment tool to measure professional competence of physiotherapy students undertaking simulation.

## Methods

### Participants

Participants in this multi-site cross-sectional study were pre-registration physiotherapy students from two Australian universities. There were 444 students in simulation and 190 students in clinical placements. The students were from years three and four of a 4-year undergraduate program and from year two of a 2-year graduate entry master program. Approval was obtained from the Human Research Ethics Committee of Curtin University (Protocol Approval HR 07/2014). Participation was voluntary and written consent was obtained from participating students and educators. All APPs were de-identified to ensure anonymity of educators and participants.

At University A, students undertook a 5-week clinical placement consisting of either 1 or 2 weeks in simulation and the subsequent weeks in clinical placement. Students were designated to particular specialty areas of cardiorespiratory, musculoskeletal or neurological physiotherapy. The APP was administered at the end of the 1 or 2 weeks of simulation and was based on the students’ entire performance in simulation over that time and therefore was a longitudinal assessment of student performance. The APP was scored by the simulation educator who was in attendance for the entire simulation period.

At University B, students undertook a 4-week simulation consisting 1 week in each three specialty areas (cardiorespiratory, musculoskeletal and neurological physiotherapy), followed by a final week of additional feedback and summative assessment activities. Student professional competency was assessed at the end of each week for the first 3 weeks by simulation educators using the APP while observing a single 25-min simulation using a standardized patient. We term this a ‘short-form assessment’ in this paper.

### Instrument

#### Assessment of physiotherapy practice

The APP is a 20-item tool used to assess professional competence in physiotherapy students. The items cover professional competence measures across the seven domains of practice which include professional behavior, communication, patient assessment, analysis and planning of interventions, performing the intervention, evidence-based practice and risk management (see Additional file 1: Appendix). Students are scored on a five-point rating scale from 0 to 4. Zero denotes that the performance indicators are infrequently/rarely demonstrated. A score of one denotes that few performance indicators are demonstrated to an adequate standard. A score of two denotes that most performance indicators for that item to be of an adequate standard. A score of three denotes most performance indicators to a good standard, and the highest score of four denotes most performance indicators to an excellent standard. Scores are summed to provide a final competence score. If all items are scored, then the student mark is out of a possible score of 80. If any items are given the rating of ‘not-assessed’, the maximal overall score is revised. Assessment forms are accompanied with examples of performance indicators and a marking rubric to improve scoring reliability. The APP as an assessment of clinical competence was designed to be implemented at the midpoint and endpoint of a clinical placement, providing a formative and summative assessment respectively. Typically, the clinical placement would be 4, 5 or 6 weeks in duration [14].

### Procedures

Simulation was undertaken on-site at each university. Experienced actors were employed as standardized patients and were trained to portray a specific patient condition and case scenario. The simulation scenarios were constructed to mimic the clinical activities undertaken in clinical placement sites such as in hospitals or private practices. The cases were reflective of representative non-critical patients requiring physiotherapy assessment and intervention. Under the guidance of an educator, students treated a variety of standardized patients throughout the day, and undertook common tasks such as reading medical files, preparing treatment plans, assessing and treating the standardized patients and documenting treatment sessions. This schedule was to reflect a typical day on a ward or in a private practice on a clinical placement. Standardized patients were trained to adopt a real patient’s medical history and, depending on the nature of the condition, were clothed in gowns with intravenous drips, drains, and connected to monitors to present in a realistic manner. Students were encouraged to interact with the standardized patients in the same manner as real patients.

In addition, APPs from students in clinical placements were also collected. These APPs represented the end of clinical unit summative scores for an entire 5 week clinical placement, scored by clinical educators.

### Rasch model

The Rasch measurement model provides a mathematical framework to explore the construct validity of an instrument. The central theory to Georg Raschs’ model is that a person having a greater ability than another person should have the greater probability of solving any item of the type in question, and similarly, one test item being more difficult than the other means that for any person the probability of solving the second test item is the greater one [18]. The unidimensionality of the instrument is evaluated by examining the goodness of fit of the items to the Rasch model [19]. To be a valid measure of student performance, the APP tool must demonstrate acceptable fit to the Rasch measurement model and display uni-dimensionality, that is, the tool measures a single construct only. The tool must present a stable and consistent hierarchical model of item difficulty, and must adequately distinguish different levels of student performance [24]. The Rasch model works by constructing interval scales from ordinal data and then identifies an item’s location along the dimension of student ability and also the item’s location with respect to other items [25].

Rasch analysis was used to evaluate the validity of the APP when administered in three different assessment formats in simulation. These include the APP following both one and two weeks of simulation (longitudinal), and the APP during a single short scenario (short-form). The APP data were also examined when administered after 5 weeks of a clinical placement.

### Data analysis

Data were analyzed with Winsteps software version 3.91.0. APPs were collected, de-identified and collated into Excel spreadsheets and imported into Winsteps for analysis.

#### Fit to the Rasch model

Uni-dimensionality is an important measure central to the Rasch model and is determined by considering several psychometric properties of the tool and its ability to fit the Rasch model. For an instrument to discriminate accurately between different levels of performance and hold internal consistency, the person separation index should be > 2.0 and the person and item reliability indexes (measuring statistical validity) would be deemed to be “very good” if > 0.91. [26]. Item functioning is explored through the infit and outfit mean square. The consistency in which educators used items, and therefore how well each item conformed to the Rasch measurement model was examined [27]. There are varying levels in the research regarding what constitutes an acceptable fit [19, 26]. For an ideal fit, the mean square value is 1.0. We considered an acceptable fit being 0.5–1.5, and values > 2.0 suggest that the item is either being used inconsistently enough to potentially corrupt the measurement model or that it is not part of the construct under examination [26]. The Infit and outfit statistics can identify items on the APP that are not consistent in their level of difficulty across all students. The items may be ambiguous, confusing or interpreted differently between scorers, or the item may not be part of the construct of clinical competence [24]. Misfitting items may require re-wording or omission.

Where rating scales are functioning satisfactorily, average measure scores for categories on the APP progress monotonically (ie those students with items rated as 3 or 4 should have higher measure scores than those with items rated 0 or 1). Additionally, to determine if each scoring category (0–4) is sufficiently defined to represent an accurate progression in achievement and define differences between performances, the Rasch-Andrich thresholds are examined. These thresholds (for example, moving from a score of zero to one or from one to two) should progress by 1.4–5 logits [26]. A logit is the unit of measurement that results when raw scores from ordinal data are transformed by Rasch modelling to log odds ratios on a common interval scale [19]. A scale with good coherence demonstrates coherence scores > 40 %. Good coherence depicts a consistent relationship between measures (the APP items) and average expected ratings (the scores from 0 to 4). A coherence score for M > C (measure implies category) demonstrates the percentage of the ratings expected to be observed in a scoring category (according to the measures) that are actually observed in the category. The C > M (category implies measure) depicts the percentage of the occurrences of the scoring category (for each 0–4) that are placed by the measures in that category [26].

#### Item hierarchy

The internal stability of items was used to determine if items progress according to a hierarchical model of difficulty and if the order of difficulty of items was appropriate and consistent for students [24]. It should be easy for students to score highly on easy items and hard to score highly on difficult items, with items in the middle ranking in a predictable manner [21]. Secondly, a comparison of the hierarchy of difficulty of items will be contrasted between the different APP assessment formats to explore consistency. Movement of more than five ranking places between item difficulties across different assessment formats was deemed to warrant further exploration of the item in the absence of any published threshold guideline for this area of analysis.

#### Differential item functioning

A scale that fits the Rasch model performs consistently irrespective of the different assessment formats and different contexts in which the tool was applied. Examining differential item functioning allows the investigation of item biases that may exist in one of these different assessment formats and contexts. In this study an analysis was established to explore differential item functioning of the APP between the three simulation formats (longitudinal 1 week, longitudinal 2 week, and short-form) and also compare these formats with the clinical placement setting. Statistically significant items with a mean difference of > 0.5 logits was considered an appropriate threshold to determine the item to be making a noticeable impact on the functioning of the scale [28]. Previous research has demonstrated, using Rasch analysis, no item bias for the APP across nine demographic variables (student and educator age, gender and experience levels, type of facility, university type, clinical area) [23] and therefore these variables were not re-examined in this study.

#### Targeting

A well targeted tool has items on the testing instrument matching the range of the students’ competency [19]. The distribution of students’ competency compared to item difficulty is examined by comparing the mean location score of students to the mean items score (set at zero). Floor or ceiling effects are explored, enabling an indication of the match between the item difficulty and the abilities of the students in the sample. Additionally, for the items in the APP to be a meaningful reflection of student performance, the student needs to be observed undertaking these skills and behaviors frequently enough to be scored by the educator. The scoring categories (0–4) also need to be used frequently enough to be meaningful. Items that are observed less frequently by educators, deeming the item to be ‘not assessed’, can cause difficulties with the scale, and suggests that the item may not be able to be appropriately assessed for the particular type of simulation format. ‘Not assessed’ is an available category for educators to choose on the APP for the item(s) they feel they have not observed sufficiently to make an informed judgement on student competency. A potential threshold for items with > 25 % of ‘not assessed’ to be considered for exclusion was explored, however, there is no established threshold from the literature regarding an acceptable percentage of missing data in a data set for valid statistical inferences [29].

## Results

A total of 1260 APPs were collected. There were 971 APPs returned from 444 students in simulation-based assessments. One week and 2 week longitudinal simulation assessments were collected from University A and included 147 and 181 APPs respectively. Additionally, there were 643 APPs from university B from short-form simulation assessments. There were 289 APPs returned from 190 students in clinical placement assessments.

### Fit to the Rasch model

Using the whole simulation sample (*n* = 971), the person separation index was 4.93, person reliability index was 0.96 and item reliability index was 0.99. Both the infit and the outfit statistics of all 20 items were < 2.0 indicating a good fit (Table 1). There was no significant disordering of step calibrations, with well-defined Rasch Andrich thresholds within the 1.4–5 logit desired range. Items 1 and 3 showed a minor step calibration disordering which can be attributed to by the low number of scores of ‘zero’ for these easier items.

**Table 1:** Rasch model individual item fit

One week					Two week					Short-form					Clinical Placement				
Item	Measure	Infit (mnsq)	Outfit (mnsq)	Number of responses (*n*)	Item	Measure	Infit (mnsq)	Outfit (mnsq)	Number of responses (*n*)	Item	Measure	Infit (mnsq)	Outfit (mnsq)	Number of responses (*n*)	Item	Measure	Infit (mnsq)	Outfit (mnsq)	Number of responses (*n*)
18	2.13	.85	.98	114	18	2.27	.71	.63	156	18	1.65	1.59	1.51	28	17	1.44	.82	.78	289
8	1.13	1.17	1.46	147	17	1.73	.72	.65	181	12	1.05	1.39	1.38	189	12	1.40	.94	.93	289
12	1.13	1.06	1.11	147	12	1.52	.79	.82	172	17	.98	.89	.85	291	10	1.17	.92	.88	288
6	1.00	.99	.90	147	10	1.42	.96	.85	179	16	.92	1.01	.98	523	18	1.14	.90	.88	280
10	.82	.75	.61	147	8	1.35	.73	.70	180	14	.88	1.11	1.10	532	11	1.09	.82	.78	289
9	.77	.47	.39	147	11	1.28	.79	.77	177	15	.49	.98	.96	532	13	.95	.87	.84	289
16	.73	1.05	.89	147	19	.94	1.05	1.09	178	10	.43	.83	.84	554	8	.76	.80	.74	289
11	.64	.73	.74	147	9	.89	.66	.63	180	7	.42	1.04	1.00	557	19	.67	.92	.89	289
17	.64	.45	.33	147	6	.78	1.07	1.18	178	11	.40	.89	.94	514	16	.51	1.02	.99	288
13	.42	.54	.54	147	16	.69	1.02	1.14	181	9	.35	.91	.90	607	9	.43	.75	.72	289
20	.31	.99	1.01	147	13	.55	.66	.66	180	19	.16	.68	.64	271	15	.13	1.03	1.00	289
7	.30	.78	.86	147	14	.38	.83	.82	180	13	.13	.85	.83	406	7	.05	.93	.93	289
14	.30	.42	.41	147	7	.08	.96	.96	181	20	-.02	.95	.97	534	14	.03	.67	.65	289
19	.09	1.60	1.54	147	15	-.03	1.06	1.12	181	8	-.10	.80	.79	507	20	.02	.96	.99	287
15	-.44	1.27	1.58	147	20	-.80	1.03	.97	181	5	-.15	1.13	1.07	601	5	-.56	1.26	1.29	289
5	−1.27	1.41	1.58	147	5	−1.41	1.22	1.33	181	4	-.43	1.36	1.38	343	6	-.62	1.20	1.20	289
3	−1.99	1.06	1.09	147	4	−2.68	1.31	1.34	181	3	−1.19	1.15	1.02	305	4	−1.43	1.26	1.29	288
4	−2.09	1.37	1.49	147	3	−2.75	1.00	1.00	181	2	−1.35	.81	.76	285	2	−2.02	1.36	1.43	289
1	−2.31	.96	.89	147	2	−2.99	1.86	1.86	181	1	−1.45	1.17	1.13	641	3	−2.30	1.32	1.34	289
2	−2.31	.96	.89	147	1	−3.19	1.03	1.04	181	6	−3.15	1.83	1.58	11	1	−2.86	1.30	1.28	289

Key: The four simulation assessment formats are presented individually. One week and Two week represent the longitudinal simulation assessment formats that were undertaken after one and two weeks of time spent in simulation respectively. Short-form assessments were those undertaken in a single 25 min exam in simulation. Clinical placement represents longitudinal assessments undertaken after a completed clinical placement in a hospital or clinic setting. Items are listed from most to least difficult for each separate assessment format. The measure score places each item along a hierarchy of difficulty. The infit and outfit scores are presented in mean-squares (mnsq) and indicate a wellness of fit of an individual item to the Rasch model. The number of times each item was scored by an educator in each format is also displayed as number of responses ‘*n*’

The data demonstrated good coherence scores for M > C (measure implies category). These were observed at 73, 79, 67, 63 and 76 % for the scores 0–4 respectively. The C > M (category implies measure) scores were 27, 79, 75, 61 and 64 % for scores 0–4 respectively.

### Item hierarchy

In the data set as a whole, items requiring clinical reasoning and those related to carrying out a physiotherapy intervention such as monitoring and progressing the intervention, as well as discharge planning and goal setting were the most difficult items on which to score highly. Items relating to professional behaviour were the easiest on which to score highly. The hierarchy of item difficulty from most difficult to least difficult is presented in Table 1.

The hierarchy of difficulty of items of the APP was reasonably consistent between assessment formats. The only significant change in order was item 6 ‘demonstrates clear and accurate documentation’ which moved to the easiest item for the short form assessments and item 8 ‘selects and measures relevant health indicators and outcomes’ which was more difficult to achieve a high scores in longitudinal simulation formats, particularly the 1 week simulation.

### Differential item functioning (DIF)

**Fig. 1:**
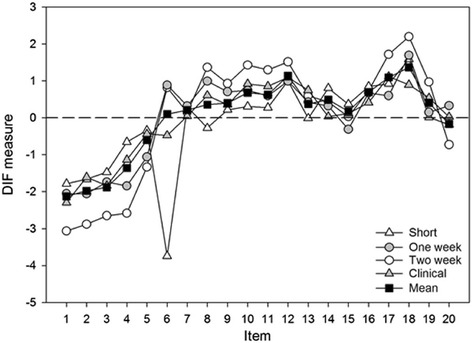
Differential Item Functioning across four assessment forms and the overall mean values of all assessment formats. The Differential Item Functioning measure maps the average item difficulty. The most difficult items score highest and the least difficult score lowest. Short refers to the single 25-min short-form simulation assessment. One week and Two week denotes the longitudinal simulation assessment formats of those time periods. Clinical represents the longitudinal assessments undertaken in clinical placements. Mean refers to an overall mean for all four assessment formats. For details of the outlier of item 6 during short form assessment, please refer to text

Item 6 ‘demonstrates clear and accurate documentation’ was the only statistically significant item to display item bias and DIF over the threshold of > 0.5 logits. This DIF occurred in the short-form simulation (Fig. 1). However, item 6 was rarely scored in this short format (*n* = 11) with educators overwhelmingly (98 % of the time) allocating this item the scoring category of ‘not-assessed’ (see Table 1).

### Targeting

**Fig. 2:**
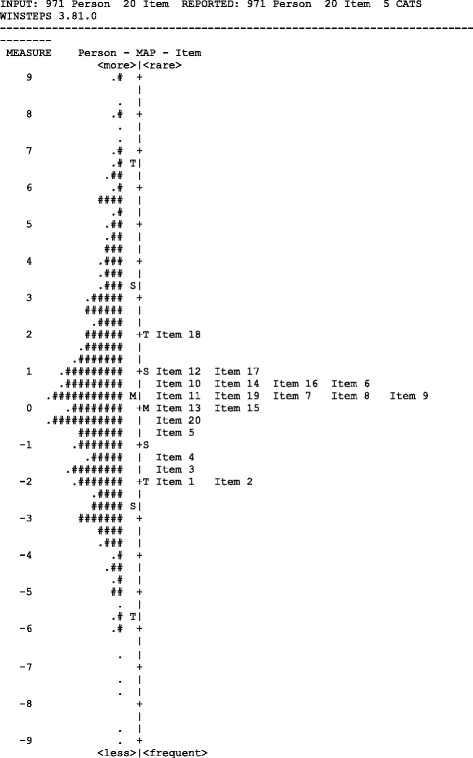
Item-Person map representing all simulation data. Person abilities (left of centre line) are mapped against item difficulty (right of centre line). Students' ability is arranged from highest performing to lowest performing, and item difficulty is from most difficult to least difficult. Each '#' represents 5 participants. Each '.' represents 1 to 4 participants. The mean student ability ('M' on left of centre line) closely matches the mean of item difficulty ('M' on right of centre line). S = 1 standard deviation, T = 2 standard deviations

Overall, the APP data from simulation demonstrated mean location scores that were obtained for students closely matching with the value of zero set for items (Fig. 2). There was no major floor or ceiling effects suggesting an overall good match of the item difficulty to student ability (Fig. 2). All longitudinal APP data (1 week and 2 weeks simulation, and clinical placement) had minimal missing or ‘non-assessed’ scores for all items. In contrast, the short-form APP data demonstrated large amounts of ‘non-assessed’ items with educators regarding many items not suitable for assessment. Nine of the 20 APP items scored more than 25 % of ‘not assessed’ (Table 1).

The APPs from the longitudinal 1 week and 2 weeks simulations demonstrated some minor floor effects. With the exception of items relating to professional conduct, there were infrequent scores of 3 or 4 on other items. There was no such floor effect on the short-form APPs. There was a minor ceiling effect on clinical placement APP scores collected after a full five weeks of clinical placement with scores of 0 not being used and a score of 1 being used infrequently.

## Discussion

This study examined the psychometric properties of the APP in simulation and found that in both longitudinal assessments, the APP was a good fit to the Rasch measurement model. In short-form simulation assessment the suitability of the tool was impeded by large amounts of non-assessed item data, indicating that students were unable to demonstrate all the skills and behaviours scored on the APP in a single assessment. These results provide evidence of sound construct validity of the APP for the examination of professional competence of physiotherapy students in longitudinal assessments of 1 and 2 weeks in simulation settings.

During both longitudinal simulation-based assessments, our data were comparable in fit to the Rasch model to our clinical placement APP data. Therefore, when used in longitudinal assessments, the APP tool performs comparably to its use in clinical environments. This demonstrates that the APP is valid in both learning environments. These results also concur with the findings of Dalton and colleagues that the APP is a valid tool for use in clinical placements, which was the original intention of the tool [14].

Both scoring categories and item concepts were well-defined for the educator and average student ability ascended with the category score as shown by the monotonical progression of Andrich thresholds. The most difficult items on which to score highly were those relating to clinical reasoning and the easiest items related to professional behaviour. These findings were similar to the results of other researchers [21, 30] who observed professional items to be scored highest, whilst problem-solving and clinical reasoning items were lowest on clinical placement assessments. The hierarchy of item difficulty was consistent between simulation assessment formats (short-form and longitudinal) with the exception of two items. Item 8 ‘selects and measures relevant health indicators and outcomes’ was scored significantly higher in the short-form assessment compared to longitudinal simulation assessments, particularly in the 1 week longitudinal format. Item 6 ‘demonstrates clear and accurate documentation’ was scored higher in the short-form assessment compared to the longitudinal formats. Item 6 was a misfit in Dalton et al. data [21], but we found this item demonstrated appropriate fit for both longitudinal assessment formats (longitudinal 1 week infit/outfit 0.99/0.90 mnsq; longitudinal 2 weeks infit/outfit 1.07/1.18 mnsq, Table 1). Additionally, items were well targeted for students, with item difficulty appropriately matched for student ability (Fig. 2). There was minimal missing or non-assessed data, which demonstrated that all 20 items may remain unchanged for assessment of student performance in longitudinal simulation.

For assessment in short-form simulation, the APP data was generally a good fit to the Rasch model for the items that educators were able to score. However, there were a large number of non-assessed items in this format, suggesting strongly that educators felt many of the APP items were not applicable in this assessment format. The short-form assessment did not appear to provide students with the opportunity to demonstrate many key clinical skills and behaviours. The use of the APP in the current form should be discouraged for use in short-form simulations until item suitability can be established. For these simulation formats to be assessed effectively, either the simulation case scenario may need to be revised to allow students to demonstrate the full range of skills demonstrating professional competence, or the APP needs to be revised, with omission of items (2–4, 6, 12, 13, 17–19) that were not able to be effectively scored in the short simulation assessment format.

There was a prevalence of lower scores generally on APP items from one and two week longitudinal simulations which caused some minor floor effects. Not surprisingly, these results were from assessments in simulation, and the simulation-based education was undertaken by students immediately prior to a further 3 or 4 weeks training on a clinical placement in the same corresponding clinical specialty area. Therefore, the scores after 1 and 2 weeks of simulation can be interpreted as providing a progress score at this stage. Educators scoring students on the APP were instructed to score students against the benchmark of new graduate performance. However, scoring against a high benchmark in early stage clinical training in a specialty area may not enable differentiation of student performance. These floor effects may also impact on student self-esteem and learning motivation if there is a mismatch between a student’s judgement of their capabilities and the lower than anticipated APP scores from simulation-based assessments [31]. Consideration may need to be given to altering the competency bench mark of APP assessments undertaken in simulation in the early stages of training in a clinical specialty area. The benchmark of ‘new graduate level’ may be more appropriate for the final APP at the end of a full 4–5 week clinical placement when students have more experience in the specialty area. Interestingly, the short-form assessments showed no floor effects despite the assessment taking place after only one week of experience in simulation. Conceivably, educators may have had differing interpretations of performance benchmarks for this short assessment format. These benchmarking concerns warrant further research in this area.

Apart from item 6 during short-form assessments, there was satisfactory DIF uniformity across the assessment formats. APP items behaved consistently and were not affected by use across different assessment formats. Whilst, item 6 in the short form assessment was isolated, this was more likely due to the low response rate of educators (*n* = 11) than true DIF discrepancies.

There are some limitations to this study. The APPs collected at both universities in simulation were used predominantly as formative feedback, to promote learning by assisting students to identify gaps between their current competencies and desired competencies. The APP scores did not contribute to the students’ overall mark for the clinical unit. The results however, informed educators of students requiring further remediation prior to undertaking subsequent clinical placements. This may have influenced the scoring intentions of the educators; however, the good model fit suggests that this was not a major issue. Additionally, we were unable to obtain a complete demographic data set for the participants in the study as data were collected from several sites and needed to be de-identified. However, previous research has not found potential co-variates, such as demographics, to impact on APP validity [21]. We were not able to brief the educators prior to the assessments about scoring objectives. The non-use of some items may have been influenced by factors beyond our control.

The approach of using standardized competency tools for assessments, that have been validated using Rasch analysis, has been previously undertaken in occupational therapy and physiotherapy [21, 32]. This study has now demonstrated the applicability of this approach to assessments also in simulation settings. This approach therefore may also be useful for consideration by other allied health professions who wish to develop their own standardized competency tools for simulation or clinical placement evaluation.

## Conclusion

When used as the assessment for longitudinal simulation, the APP instrument demonstrated construct validity and accurately measured physiotherapy students’ professional competence. This is an important finding due to the increased nature of this form of assessment in physiotherapy education. However, when used in a single short-form assessment during simulation, a revised version of the APP may be required due to many items not being applicable to score. Physiotherapy educators who assess students during simulation using a single ‘one-off’ clinical assessment need to be mindful of the applicability of tools, such as the APP, as the validity is not fully established.

## Abbreviations

ANTS, anesthetists non-technical skills; APP, assessment of physiotherapy practice; DIF, differential item functioning; NOTECHS, non-technical skills evaluation tool; OSCE, objective structured clinical exam

## Additional file

Additional file 1: Appendix. The Assessment of Physiotherapy Practice. The mark sheet for the Assessment of Physiotherapy Practice and accompanying examples of performance indicators. (PDF 54 kb)
