# Supplementary material for: The validity of a professional competence tool for physiotherapy students in simulation-based clinical education: a Rasch analysis
Source: BMC Med Educ. 2016 Aug 5;16:196. doi: 10.1186/s12909-016-0718-x (PMC4974700; doi:10.1186/s12909-016-0718-x)
Supplement: Additional file 1: — Appendix. The Assessment of Physiotherapy Practice. The mark sheet for the Assessment of Physiotherapy Practice and accompanying examples of performance indicators. (PDF 54 kb) [file 12909_2016_718_MOESM1_ESM.pdf]

Assessment of Physiotherapy Practice
End of Unit
Summative Assessment

Student name:.. **Student No:**……………………**Days Absent:**................................

Facility/Experience:………………………………………………………………………………**Date**:...

Educator/s Name & Signature:………………………………………………………....**Student Signature**:…………….......................

0 = Infrequently/rarely demonstrates performance indicators
1 = Demonstrates few performance indicators to an adequate standard
2 = Demonstrates most performance indicators to an adequate standard
3 = Demonstrates most performance indicators to a good standard
4 = Demonstrates most performance indicators to an excellent standard
not assessed = item was not assessed
***Note.* a rating of 0 or 1 indicates that a minimum acceptable standard has not been achieved**

**Professional Behaviour**	**Circle one number only**					
1. Demonstrates an understanding of patient/client rights and consent	0	1	2	3	4	not assessed
2. Demonstrates commitment to learning	0	1	2	3	4	not assessed
3. Demonstrates ethical, legal & culturally sensitive practice	0	1	2	3	4	not assessed
4. Demonstrates teamwork	0	1	2	3	4	not assessed
**Communication**						
5. Communicates effectively and appropriately - Verbal/non-verbal	0	1	2	3	4	not assessed
6. Demonstrates clear and accurate documentation	0	1	2	3	4	not assessed
**Assessment**						
7. Conducts an appropriate patient/client interview	0	1	2	3	4	not assessed
8. Selects and measures relevant health indicators and outcomes	0	1	2	3	4	not assessed
9. Performs appropriate physical assessment procedures	0	1	2	3	4	not assessed
**Analysis & Planning**						
10. Appropriately interprets assessment findings	0	1	2	3	4	not assessed
11. Identifies and prioritises patient's/client's problems	0	1	2	3	4	not assessed
12. Sets realistic short and long term goals with the patient/client	0	1	2	3	4	not assessed
13. Selects appropriate intervention in collaboration with patient/client	0	1	2	3	4	not assessed
**Intervention**						
14. Performs interventions appropriately	0	1	2	3	4	not assessed
15. Is an effective educator	0	1	2	3	4	not assessed
16. Monitors the effect of intervention	0	1	2	3	4	not assessed
17. Progresses intervention appropriately	0	1	2	3	4	not assessed
18. Undertakes discharge planning	0	1	2	3	4	not assessed
**Evidence-based Practice**						
19. Applies evidence based practice in patient care	0	1	2	3	4	not assessed
**Risk Management**						
20. Identifies adverse events/near misses and minimises risk associated with assessment and interventions	0	1	2	3	4	not assessed

In your opinion as a clinical educator, the overall performance of this student in the clinical unit was:

Not adequate ☐ Adequate ☐ Good ☐ Excellent ☐

Scoring rules:
- ✓ Circle not assessed only if the student has not had an opportunity to demonstrate the behaviour
- ✓ If an item is not assessed it is not scored and the total APP score is adjusted for the missed item.
- ✓ Circle only one number for each item
- ✓ If a score falls between numbers on the scale the higher number will be used to calculate a total.
- ✓ Evaluate the student's performance against the minimum standard expected for a beginning/entry level physiotherapist.

Dalton M., Keating J., Davidson M. (2009) Development of the Assessment of Physiotherapy Practice (APP): A standardised and valid approach to assessment of clinical competence in physiotherapy. Australian Learning and Teaching Council (ALTC) Final report PP6-28. http://www.altc.edu.au/project-development-clinical-assessment-2006

Examples of Performance Indicators

Professional Behaviour

1. Demonstrates an understanding of patient/client rights and consent

- informed consent is obtained and recorded according to protocol
- understands and respects patients'/clients' rights
- allows sufficient time to discuss the risks and benefits of the proposed treatment with patients/clients and carers
- refers patients/clients to a more senior staff member for consent when appropriate
- advises supervisor or other appropriate person if a patient/client might be at risk
- respects patients'/clients' privacy and dignity
- maintains patient/client confidentiality
- applies ethical principles to the collection, maintenance, use and dissemination of data and information

2. Demonstrates commitment to learning

- responds in a positive manner to questions, suggestions &/or constructive feedback
- reviews and prepares appropriate material before and during the placement
- develops and implements a plan of action in response to feedback
- seeks information/assistance as required
- demonstrates self-evaluation, reflects on progress and implements appropriate changes based on reflection
- takes responsibility for learning and seeks opportunities to meet learning needs
- uses clinic time responsibly

3. Demonstrates ethical, legal & culturally sensitive practice

- follows policies & procedures of the facility
- advises appropriate staff of circumstances that may affect adequate work performance
- observes infection control, and workplace health and safety policies
- arrives fit to work
- arrives punctually and leaves at agreed time
- calls appropriate personnel to report intended absence
- wears an identification badge & identifies self
- observes dress code
- completes projects/tasks within designated time frame
- maintains appropriate professional boundaries with patients/clients and carers
- demonstrates appropriate self-care strategies (eg stress management)
- acts ethically and applies ethical reasoning in all health care activities
- Practises sensitively in the cultural context
- acts within bounds of personal competence, recognizing personal and professional strengths and limitations

4. Demonstrates teamwork

- demonstrates understanding of team processes
- contributes appropriately in team meetings
- acknowledges expertise and role of other health care professionals and refers/liaises as appropriate to access relevant services
- advocates for the patient/client when dealing with other services
- collaborates with the health care team and patient/client and to achieve optimal outcomes
- cooperates with other people who are treating and caring for patients/clients
- works collaboratively and respectfully with support staff

Communication

5. Communicates effectively and appropriately - Verbal/non-verbal

- greets others appropriately
- questions effectively to gain appropriate information
- listens carefully and is sensitive to patient/client and carer views
- respects cultural and personal differences of others
- gives appropriate, positive reinforcement
- provides clear instructions
- uses suitable language & avoids jargon
- demonstrates an appropriate range of communication styles (eg patients/clients, carers, administrative and support staff, health professionals, care team)
- recognises barriers to optimal communication
- uses a range of communication strategies to optimize patient/client rapport and understanding (eg hearing impairment, non-English speaking, cognitive impairment, consideration of non-verbal communication)
- appropriately uses accredited interpreters
- maintains effective communication with clinical educators
- actively explains to patients/clients and carers their role in care, decision-making and preventing adverse events
- actively encourages patients/clients to provide complete information without embarrassment or hesitation
- communication with patient/client is conducted in a manner and environment that demonstrates consideration of confidentiality, privacy and patient's/client's sensitivities
- negotiates appropriately with other health professionals

6. Demonstrates clear and accurate documentation

- writes legibly
- completes relevant documentation to the required standard (eg., patient/client record , statistical information, referral letters)
- maintains records compliant with legislative medico-legal requirements
- complies with organisational protocols and legislation for communication
- adapts written material for a range of audiences (e.g. provides translated material for non-English speaking people, considers reading ability, age of patient/client)

Assessment

7. Conducts an appropriate patient/client interview

- positions person safely and comfortably for interview
- structures a systematic, purposeful interview seeking qualitative and quantitative details
- asks relevant and comprehensive questions
- politely controls the interview to obtain relevant information
- responds appropriately to important patient/client cues
- identifies patient's/client's goals and expectations
- conducts appropriate assessment with consideration of biopsychosocial factors that influence health.
- seeks appropriate supplementary information, accessing other information, records, test results as appropriate and with patient's/client's consent
- generates diagnostic hypotheses, identifying the priorities and urgency of further assessment and intervention
- completes assessment in acceptable time

8. Selects and measures relevant health indicators and outcomes

- selects <u>all</u> appropriate variable/s to be measured at <u>baseline</u> from WHO ICF domains of impairment, activity limitation and participation restriction.
- identifies and justifies variables to be measured to <u>monitor</u> treatment response and outcome.
- selects appropriate <u>tests/outcome measures</u> of each variable for the purpose of diagnosis, monitoring and outcome evaluation.
- links outcome variables with treatment goals
- communicates the treatment evaluation process and outcomes to the client
- identifies, documents and acts on factors that may compromise treatment outcomes

9. Performs appropriate physical assessment procedures

- considers patient/client comfort and safety
- respects patient's/client's need for privacy and modesty (eg provides draping or gown)
- structures systematic, safe and goal oriented assessment process accommodating any limitations imposed by patient's/client's health status
- plans assessment structure and reasoning process using information from patient/client history and supportive information
- demonstrates sensitive and appropriate handling during the assessment process
- applies all tests and measurements safely, accurately and consistently
- sensibly modifies assessment in response to patient/client profile, feedback and relevant findings
- appropriate tests are performed to refine diagnosis
- assesses/appraises work, home or other relevant environments as required
- completes assessment in acceptable time

Examples of Performance Indicators

Analysis & Planning

10. Appropriately interprets assessment findings

- describes the implications of test results
- describes the presentation and expected course of common clinical conditions
- relates signs and symptoms to pathology
- relates signs symptoms and pathology to environmental tasks and demands
- interprets findings at each stage of the assessment to progressively negate or reinforce the hypothesis/es
- makes justifiable decisions regarding diagnoses based on knowledge and clinical reasoning
- prioritises important assessment findings
- compares findings to normal

11. Identifies and prioritises patient's / client's problems

- generates a list of problems from the assessment
- justifies prioritisation of problem list based on knowledge and clinical reasoning
- collaborates with the patient/client to prioritise the problems
- considers patient's/clients values, priorities and needs

12. Sets realistic short and long term goals with the patient / client

- negotiates realistic short treatment goals in partnership with patient/client
- negotiates realistic long treatment goals in partnership with patient/client
- Formulates goals that are specific, measurable, achievable and relevant, with specified timeframe
- considers physical, emotional and financial costs and relates them to likely gains of physiotherapy intervention

13. Selects appropriate intervention in collaboration with the patient / client

- engages with patient/client to explain assessment findings, discuss intervention strategies and develop an acceptable plan
- options for physiotherapy intervention are identified and justified, based on patient/client needs, on best evidence and available resources
- considers whether physiotherapy treatment is indicated
- demonstrates a suitable range of skills and approaches to intervention
- describes acceptable rationale (eg likely effectiveness) for treatment choices
- balances needs of patients/clients and care givers with the need for efficient and effective intervention
- demonstrates understanding of contraindications and precautions in selection of intervention strategies
- advises patient/client about the effects of treatment or no treatment

Intervention

14. Performs interventions appropriately

- considers the scheduling of treatment in relation to other procedures eg medication for pain, wound care.
- demonstrates appropriate patient/client handling skills in performance of interventions
- performs techniques at appropriate standard
- minimizes risk of adverse events to patient/client and self in performance of intervention (including observance of infection control procedures and manual handling standards)
- prepares environment for patient/client including necessary equipment for treatment
- identifies when group activity might be an appropriate intervention
- demonstrates skill in case management
- recognises when to enlist assistance of others to complete workload
- completes intervention in acceptable time
- refers patient/client on to other professional/s when physiotherapy intervention is not appropriate, or requires a multi-disciplinary approach

15. Is an effective educator / health promoter

- demonstrates skill in patient/client education eg modifies approach to suit patient/client age group, uses principles of adult learning
- demonstrates skills in conducting group sessions
- a realistic self-management program for prevention and management is developed with the patient/client
- provides information using a range of strategies that demonstrate consideration of patient/client needs
- confirms patient's/client's or caregivers understanding of given information
- uses appropriate strategies to motivate the patient/client and caregiver to participate and to take responsibility for achieving defined goals
- discusses expectations of physiotherapy intervention and its outcomes
- provides feedback to patient/client regarding health status
- educates the patient/client in self evaluation
- encourages and acknowledges achievement of short and long term goals

16. Monitors the effects of intervention

- incorporates relevant evaluation procedures/outcome measures within the physiotherapy plan
- monitors patient/client throughout the intervention
- makes modifications to intervention based on evaluation
- records and communicates outcomes where appropriate

17. Progresses intervention appropriately

- demonstrates &/or describes safe and sensible treatment progressions
- modifications, continuation or cessation of intervention are made in consultation with the patient/client, based on best available evidence
- discontinues treatment in the absence of measurable benefit

18. Undertakes discharge planning

- begins discharge planning in collaboration with the health care team at the time of the initial episode of care
- describes strategies that may be useful for maintaining or improving health status following discharge
- arranges appropriate follow-up health care to meet short and long term goals
- addresses patient/client and carer needs for ongoing care through the coordination of appropriate services

Evidence Based Practice

19. Applies evidence based practice in patient care

- considers the research evidence, patient/client preferences, clinical expertise and available resources in patient/client management
- locates and applies relevant current evidence eg., clinical practice guidelines and systematic reviews
- assists patients/clients and carers to identify reliable and accurate health information
- shares new evidence with colleagues
- participates in quality assessment procedures when possible

Risk Management

20. Identifies adverse events and near misses and minimises risk associated with assessment and interventions

- monitors patient/client safety during assessment and treatment.
- complies with workplace guidelines on patient/client handling
- complies with organizational health and safety requirements
- describes relevant contraindications and precautions associated with assessment and treatment
- reports adverse events and near misses to appropriate members of the team
- implements appropriate measures in case of emergency
- reports inappropriate or unsafe behaviour of a co-worker or situations that are unsafe
